# Immunological and homeostatic pathways of alpha -1 antitrypsin: a new therapeutic potential

**DOI:** 10.3389/fimmu.2024.1443297

**Published:** 2024-08-19

**Authors:** Carmen Mazzuca, Laura Vitiello, Silvia Travaglini, Fatima Maurizi, Panaiotis Finamore, Simona Santangelo, Amelia Rigon, Marta Vadacca, Silvia Angeletti, Simone Scarlata

**Affiliations:** ^1^ Unit of Internal Medicine and Geriatrics, Respiratory Pathophysiology and Thoracic Endoscopy, Fondazione Policlinico Campus Bio Medico University Hospital- Rome, Rome, Italy; ^2^ Pediatric Allergology Unit, Bambino Gesù Children's Hospital IRCCS, Rome, Italy; ^3^ Department of Human Sciences and Promotion of the Quality of Life, San Raffaele University, Rome, Italy; ^4^ Clinical and Research Section of Rheumatology and Clinical Immunology, Fondazione Policlinico Universitario Campus Bio-Medico, Rome, Italy; ^5^ Unit of Clinical Laboratory Science, University Campus Bio-Medico of Rome, Rome, Italy

**Keywords:** serine protease inhibitor superfamily (SERPIN), anti-inflammation, autoimmune disease, infectious disease, Severe Acute Respiratory Syndrome Coronavirus2 (SARS-CoV-2)

## Abstract

α -1 antitrypsin (A1AT) is a 52 kDa acute-phase glycoprotein belonging to the serine protease inhibitor superfamily (SERPIN). It is primarily synthesized by hepatocytes and to a lesser extent by monocytes, macrophages, intestinal epithelial cells, and bronchial epithelial cells. A1AT is encoded by SERPINA1 locus, also known as PI locus, highly polymorphic with at least 100 allelic variants described and responsible for different A1AT serum levels and function. A1AT inhibits a variety of serine proteinases, but its main target is represented by Neutrophil Elastase (NE). However, recent attention has been directed towards its immune-regulatory and homeostatic activities. A1AT exerts immune-regulatory effects on different cell types involved in innate and adaptive immunity. Additionally, it plays a role in metal and lipid metabolism, contributing to homeostasis. An adequate comprehension of these mechanisms could support the use of A1AT augmentation therapy in many disorders characterized by a chronic immune response. The aim of this review is to provide an up-to-date understanding of the molecular mechanisms and regulatory pathways responsible for immune-regulatory and homeostatic activities of A1AT. This knowledge aims to support the use of A1AT in therapeutic applications. Furthermore, the review summarizes the current state of knowledge regarding the application of A1AT in clinical and laboratory settings human and animal models.

## Highlights

- α-1antitrypsin has anti-inflammatory and immunomodulatory properties.
- α-1antitrypsin has the ability to regulate the metabolism of metals and lipids.
- *In vivo* models of enzyme deficiency demonstrate the therapeutic potential of α-1antitrypsin in modulating immune system.
- α-1antitrypsin is a potential treatment option for pulmonary, autoimmune, and infectious diseases.

## 1 Introduction

α -1 antitrypsin (A1AT) (Alpha-1 Proteinase Inhibitor, α1-Pi) belongs to the serine protease inhibitor superfamily (SERPIN). A1AT is a 52 kDa acute-phase glycoprotein and it is encoded by SERPINA1 locus, also known as Pi locus, located on the long arm of chromosome 14 (14q31-32.3). It exhibits significant polymorphism, to date at least 100 allelic variants of A1AT have been described. The classification is based on their phenotypic expression and isoelectric mobility during isoelectric focusing; the three most common phenotypes are: PiM (medium), PiS (slow), and PiZ (very slow) (1). Among these variants, the M- type allele is considered the common and functional form of A1AT. Homozygosis for the M-type allele results in the PiMM phenotype, which represents the well- working functioning protein phenotype. In contrast, some alleles lead to reduced synthesis or dysfunctional protein production, or both.

Severe A1AT deficiency (A1ATD) is associated with specific variants, such as Z- or S- types, which can result in the early onset and sever development of emphysema, liver disease, and, rarely, multiorgan vasculitis and necrotizing panniculitis (2).

A1AT is abundant in the plasma with a mean concentration of 1.3 g/L and a plasma half-life of 4-5 days. It is primarily produced by hepatocytes, although minor amounts are also synthesized by monocytes, macrophages, intestinal epithelial cells, and bronchial epithelial cells (3). It acts as an inhibitor of various serine proteinases, including proteinase 3 (PR3) (a potent elastase produced by neutrophils), cathepsin G, plasmin activator, thrombin, trypsin and chymotrypsin. It is primary target is Neutrophil Elastase (NE) (4), a serine protease released by activated neutrophils during inflammation. Its elastolytic burden can cause damage to the structural components of the wall and lead to lung tissue injury and destruction if not properly balanced by A1AT activity (5).

A1ATD is primarily characterized by two clinical conditions resulting from a pathophysiological mechanism that leads to either gain or loss of function defects. This occurs due to the polymerization of the protein A1AT and its subsequent accumulation in the endoplasmic reticulum of hepatocytes, which causes liver damage and lung dysfunction. In the lungs, the loss of A1AT’s antiprotease function against NE, caused by a significant reduction in A1AT serum levels, results in the destruction of emphysematous lung tissue. This imbalance between proteinase and antiproteinase, along with the increased inflammation observed in A1ATD (mediated by LTB4, CXCL8, and TNFα), is the primary mechanism leading to lung disease. In addition to the liver and lung issues mentioned, there is also a link between the A1AT PI Z variant and antineutrophil cytoplasmatic antibody (ANCA)- associated vasculitis. The antiproteinase-3 (C-ANCA) marker is highly sensitive and specific for diagnosing granulomatosis with polyangiitis, GPA. C-ANCA recognizes and reduces PR3 proteolytic activity and can interfere with the PR3/A1AT bond. It has been suggested that the reduced inhibition of PR3 due to A1ATD may lead to increased production of PR3-directed C-ANCA autoantibodies. Additionally, A1AT may have a diminished ability to bind PR3 and counteract its proteolytic effect on vessels. A rare form of panniculitis related to A1ATD also exists, occurring in 1 in 1,000 individuals with the PI ZZ genotype. It has been proposed that the PI Z variant may aggregate into polymers that precipitate and accumulate in cutaneous soft tissues, leading to inflammation and necrotizing skin lesions (6, 7)

**Figure 1:**
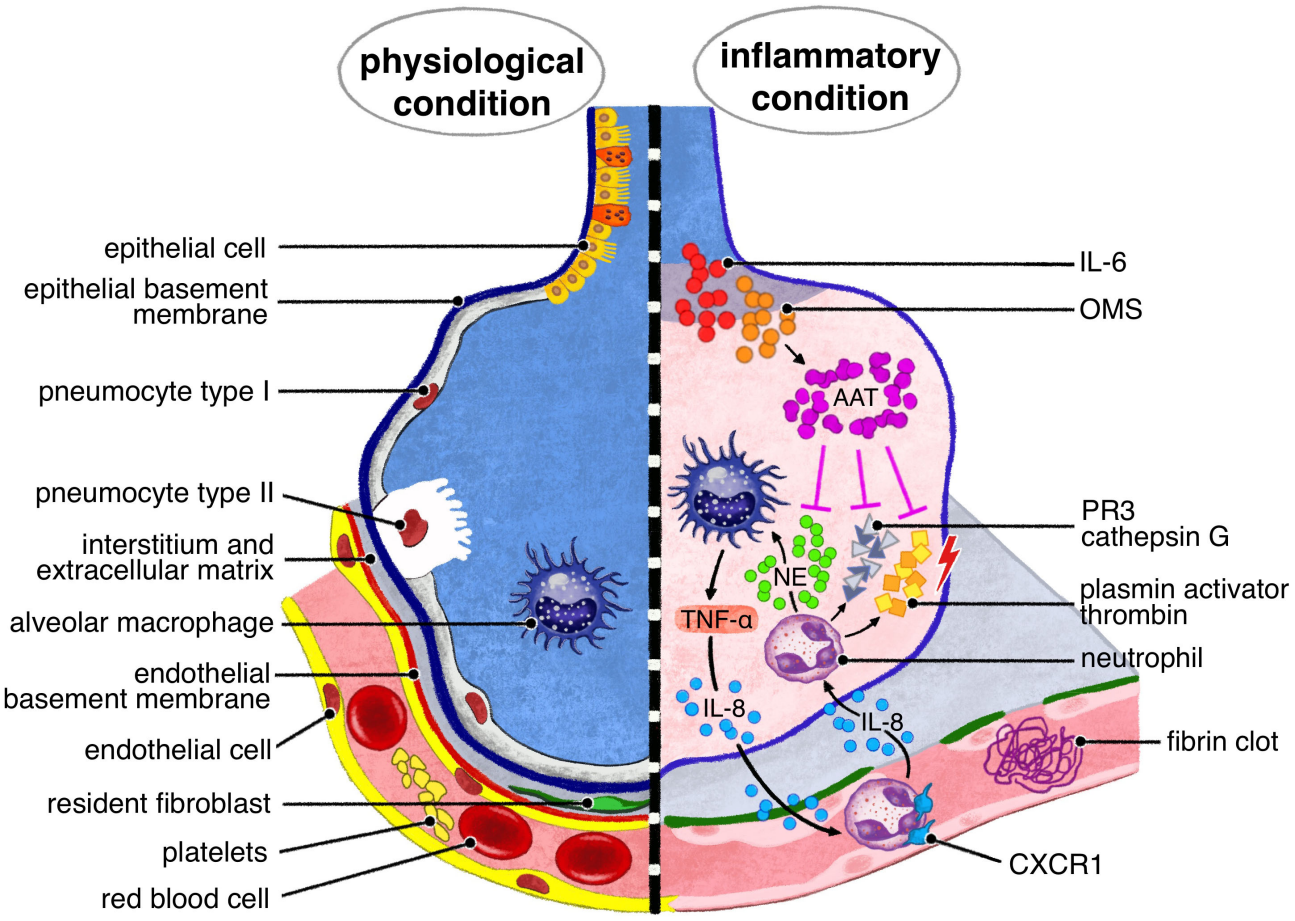
A1AT pulmonary production is due to tissue macrophages and bronchial epithelial cells, under IL 6, oncostatin M stimuli. The main target of A1AT is neutrophil elastase, NE, released by neutrophils recruited by IL -8. Other serin- proteases, responsible of lung tissue injury, target of A1AT are proteinase 3 (PR3), cathepsin G, plasmin activator and thrombin.

A1AT is the most abundant serum serine protease inhibitor with anti-protease property (8) but it also exhibits immune-regulatory activities. Several studies suggest that A1AT carries out these actions independently of each other (9). Under normal conditions, A1AT expression is regulated by promoters, while during inflammatory responses, it is stimulated by various factors. Its expression is modulated by enhancers, like interleukin-6 (IL-6) and related to cytokines (e.g. oncostatin M, OSM) (**Figure 1**) (10). A1AT is now widely recognized as an immunomodulatory factor, exerting its effect on different cell types involved in both innate and adaptive immunity, primarily by dampening inflammation. Emerging evidence suggests that A1AT is involved in regulating metal and lipid metabolism. These alternative biological effects have sparked speculation regarding its potential use in A1AT augmentation therapy, as still observed in a range of conditions.

Data on the safety of A1AT replacement therapy remain limited due to the rarity of the underlying condition, which currently has only one approved indication for treatment. Nonetheless, evidence from randomized clinical trials comparing A1AT therapy to placebo indicates a strong safety and tolerability profile. Over 90% of reported side effects were mild or moderate, primarily including respiratory tract infections, gastrointestinal issues, and general constitutional disorders. The incidence of adverse events (AEs) was 28.9% in the treatment group, with serious adverse events (SAEs) occurring in 26.3% of cases, showing no statistically significant difference compared to the control group. Further supporting this safety profile, the RAPID study reconfirmed these findings, reporting that only one patient (1%) experienced a severe adverse event leading to study dropout and subsequent death from respiratory failure. These results underscore the relative safety of A1AT replacement therapy, despite the severity of the underlying condition and the limited population of patients receiving this treatment. Existing studies, including those comparing the treatment with placebo, have focused primarily on evaluating the general safety and tolerability of the treatment, reporting predominantly mild or moderate adverse effects. There have been no significant increases in the incidence of opportunistic infections or reactivation of autoimmune diseases in patients undergoing A1AT replacement therapy. Continued monitoring and additional studies are essential to fully understand the long-term safety and efficacy of this therapy (11, 12).

In this review, we provide an up-to-date understanding of the molecular mechanisms and regulatory pathways involved in A1AT immunomodulatory actions, as well as its homeostatic functions in metal and lipid metabolism. Furthermore, we present a summary of the existing knowledge on the application of A1AT in human clinical settings (**Table 1**) and laboratory experiments on animal models (**Table 2**).

**Table 1:** Human model.

AUTHOR, YEAR	DISEASE	TREATMENT	RESULTS
Mcelvaney et al., 1991 (13)	Cystic Fibrosis	Aerosol therapy	Complete inhibition of NE activity within respiratory epithelial lining fluid
Griese et al., 2007 (14)	Cystic Fibrosis	Aerosol therapy	After 4 weeks, decrease of NE activity, neutrophil count, levels of pro-inflammatory cytokines (IL8, IL1beta, TNF alpha, LTB4) in sputum
Martin et al., 2006 (15)	Cystic Fibrosis	Aerosol therapy	A phase II trial on 39 patients: reduction in NE activity and sputum NE/AAT complex and MPO levels; clinically the treatment showed an improvement in number of exacerbations in absence of adverse effect
Eden e et al., 2003 (16)	Asthma	–	Data from the National Heart, Lung, and Blood Institute Registry on 1052 subjects with A1ATD showed no more efficacy of the augmentation treatment in reducing FEV1 decline in the asthma groups compared to asthma-free groups; the subgroup analysis showed better efficacy in the FEV1 35 to 49% category
Blanco I et al., 2008 (17)	Severe Persistent Asthma	Intravenous administration of hA1AT	Case report about a 27-year-old woman affected by Widal triad with a severe and resistant asthma. The treatment significantly reduced the number of emergency consultations and hospital admissions, it decreased the need for steroid therapy and it progressively improved the quality of life.
Dowd s.k et al., 1995 (18)	Chronic cutaneous vasculitis-A1ATD associated	Intravenous administration of hA1AT	Case report about a 49-year-old white man with A1AT deficiency affected by chronic cutaneous vasculitis refractory to colchicine and prednisone. The augmentation treatment totally resolved the cutaneous lesions within 48 hours.
Giannoni l et al., 2020 (19)	Steroid- resistant gastrointestinal GVHD	Intravenous administration of hA1AT	16 patients in advanced-stage gut SR-GVHD treated with hA1AT: overall response rate (ORR) was 44%, with a complete response (CR) rate of 27%, gastrointestinal response was observed in 61% of patients, with a median time to best response was 21 days.
Marcondes m et al., 2016 (20)	Steroid-Refractory Acute GVHD (SR-aGVHD)	Intravenous administration of hA1AT	A phase I/II open-label single-center study:12 patients with SR-aGVHD who received A1AT as salvage therapy. Clinical manifestation improved in 8 of 12 subjects of which 4 had a complete response, in absence of relevant toxicities
Magenau jm et al., 2018 (21)	Steroid-Refractory Acute GVHD (SR-aGVHD)	Intravenous administration of hA1AT	A multicenter clinical study: forty patients with SR-aGVHD received intravenous A1AT as first-line treatment. By day 28 the 35% of subjects had a complete response and at day 60 responses were sustained in 73% without immunosuppression.

**Table 2:** Animal model.

AUTHOR, YEAR	DISEASE	MODEL	TREATMENT	RESULTS
Churg et al., 2003 (22)	Cigarette smoke-induced emphysema	Mice model	Intraperitoneal administration of prolastin	After 6 months, treatment provided 63% protection against increased airspace size and it decreased neutrophils and macrophages in BAL, approximately 75% and 50%
Churg et al., 2007 (23)	Cigarette smoke-induced emphysema	Mice model	Intraperitoneal injection of prolastin,	Reduction of smoke-induced production and release of macrophage metalloelastase (mmp-12) and TNF-alpha, in a dose response fashion
Kakimoto k et al., 1995 (24)	Collagen-induced arthritis (CIA)	Rats and mice models	Ono-5046, a specific neutrophil elastase inhibitor	The treatment suppressed the development and severity of CIA in animal models.
Grimstein c et al., 2011 (25)	Collagen-induced arthritis (CIA)	Mice model	hA1AT therapy vs recombinant adeno-associated virus (raav8)-mediated gene therapy	Both treatments significantly delayed onset and ameliorated disease development of arthritis in CIA mouse model.
Cantin am et al., 1999 (26)	Chronic pseudomonas aeruginosa lung infection	Mice model	Aerosol therapy	Aerosolized prolastin significantly decreased elastase activity, lung neutrophil counts and bacterial colony counts.
Zhang et al., 2006 (27)	Type 1 diabetes	Mice model	Intramuscular injection of recombinant adeno-associated virus (raav1)-mediated gene therapy	A1at gene therapy attenuated cell-mediated autoimmunity, it altered the t cell receptor repertoire, and it efficiently prevented type 1 diabetes in the nonobese diabetic mouse model.
Lewis e c et al., 2005 (28)	Type 1 diabetes in nonobese diabetic (nod) mice	Mice model	Injection of hA1AT	Haat monotherapy prolonged islet graft survival and normoglycemia in transplanted allogenic diabetic mice, lasting until the development of anti-haat antibodies
Pileggi a et al., 2008 (29)	Type 1 diabetes in nonobese diabetic (nod) mice	Mice model	Intraperitoneal prolastin	Haat monotherapy achieved a prolongation of islet allograft survival in the stringent autoimmune diabetic nod mouse model.

To gather the most current knowledge, we conducted a review of the literature using PUBMED, Google Scholar and the Cochrane Library Databases. Our search strategy utilized appropriate generic terms to ensure a comprehensive coverage of relevant studies.

## 2 Discussion

### 2.1 Anti-inflammatory and immunomodulatory properties of A1AT

**Figure 2:**
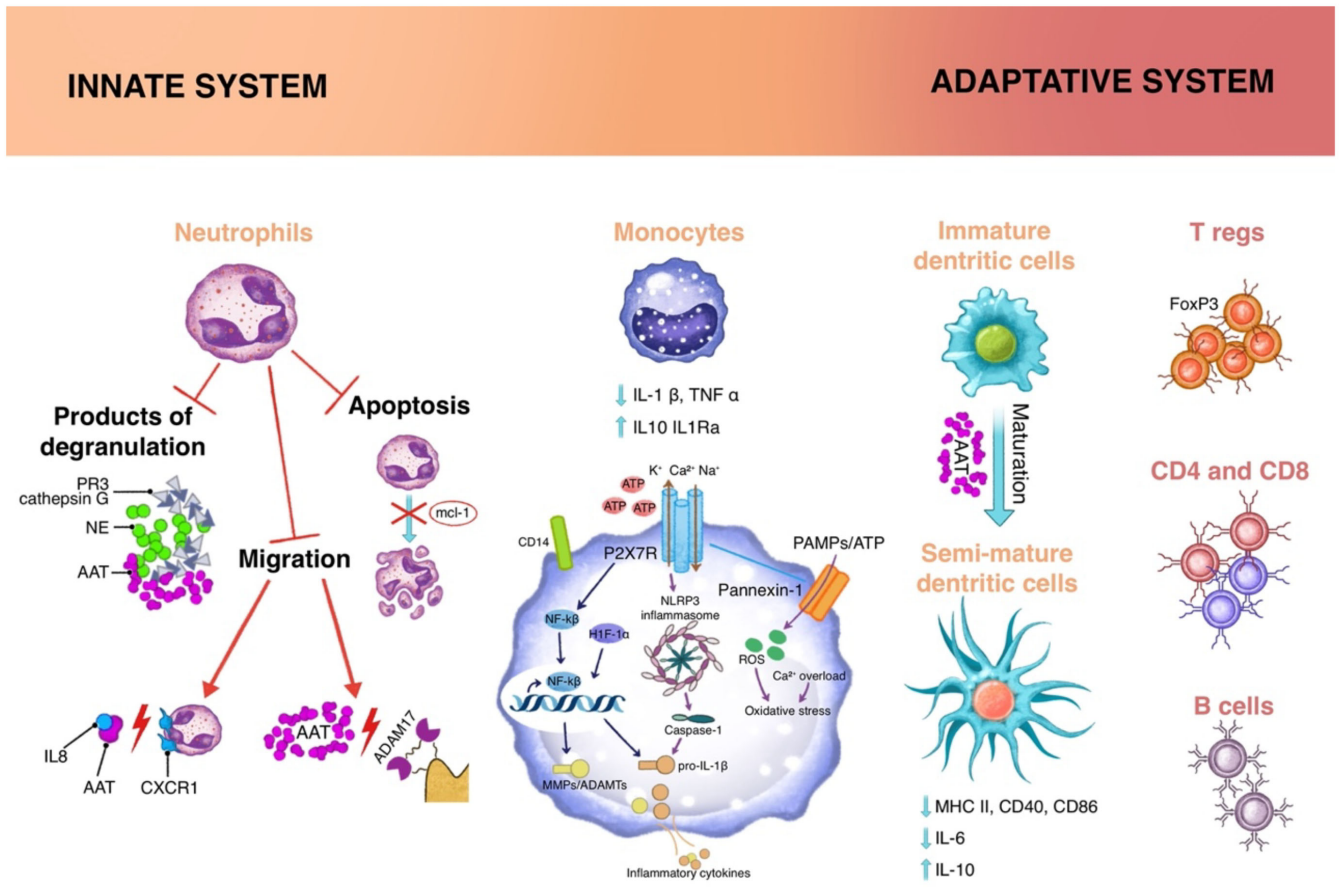
Immunomodulatory functions of A1AT acts on different cell types, both of innate and adaptive immunity. Innate cells: Neutrophils are involved through inhibitory mechanisms on the degradation products as well as tissue migration process and cellular apoptosis; the cytokine milieu influences Monocytes through different mechanisms: 1) impairing of the ATP-gated P2X7 receptor, that activates the NLPR3 inflammasome; 2) increasing expression of Nf-κB inhibitor IκB and reducing translocation of Nf-κB to the nucleus; 3) regulating CD14 expression on cellular membrane; Dendritic cells, at the interface between innate and adaptive immunity, in the presence of A1AT, present a semi-mature profile, in which there is a reduction in the expression of MHC II, CD40, CD86. Adaptative cells: the extracellular milieu and semimature dendritic cells reduce the activation of effector cells and sustain the proliferation of regulatory T cells (Tregs).

Several studies, both in preclinical models and in clinical trials, have been conducted to verify whether A1AT could be exploited in chronic inflammation diseases (25, 30). Interestingly enough, some reports evidence a role in sustaining immune response to pathogens (31) and an enhancer effect on secretion of lipopolysaccharide (LPS)-induced tumor necrosis factor-alpha (TNF α), interleukin (IL)-6, and IL-8 from monocytes and neutrophils (32). This apparent contradiction can be explained as, being by the fact that A1AT is an acute phase protein and A1AT can have opposing effects depending on the cytokine milieu and, above all, on the time of their release during the inflammation. As described by Janciauskiene and colleagues, short term stimulation of monocytes and neutrophils with LPS and A1AT results in a higher release of proinflammatory cytokines compared to stimulation with LPS alone; conversely, 18 hours stimulation with LPS in the presence of A1AT induces an increase in the production of the anti-inflammatory cytokine IL-10 (8). However, most of the literature highlights the immunosuppressive and protolerogenic effects of A1AT. In the following sections, we will briefly explore the activity of A1AT on innate and adaptive immunity (**Figure 2**).

#### 2.1.1 Effects on innate immune system

Neutrophils are the most abundant leukocytes population in peripheral blood, and they exert their activity early during inflammation, releasing cytokines, proteases, producing reactive oxygen species (ROS), and creating neutrophils extracellular traps (NETs) (33) Neutrophils represent the main targets of A1AT action: being an anti-protease, its primary effect is to inhibit serine proteases deriving from degranulating neutrophils including NE, cathepsin G and PR3 (34). A1AT can impair neutrophil migration towards inflamed tissues through two different mechanisms, affecting both IL-8-induced chemotaxis and chemotaxis induced by soluble immune complexes (sIC) (35). A1AT binds IL-8, and the resulting complex is not able to engage the CXCR1 receptor (IL-8 receptor) and inhibits downstream calcium flux and cytoskeleton rearrangements. At the same time, A1AT can associate to lipid rafts on neutrophils membrane, where it can interact with disintegrin/metalloproteinase 17 (ADAM-17), a membrane protein that controls the shedding of FCγRIIb (CD16b), an event a process that is necessary for neutrophils chemotaxis (35).

Alpha-1-antitrypsin (A1AT), beyond its role as a serine protease inhibitor, exhibits significant antioxidative properties, playing a crucial role in the neutralization of reactive oxygen species (ROS) through sophisticated mechanisms. A1AT contains methionine residues that are highly susceptible to oxidation by ROS, particularly singlet oxygen and hydroxyl radicals, resulting in the formation of methionine sulfoxide. This oxidation not only neutralizes ROS but also prevents the methionine residues from inducing further cellular damage. Methionine sulfoxide can be subsequently reduced back to methionine by specific reductases, thereby restoring A1AT’s functionality and enabling continuous ROS scavenging (4, 36).

Beside the described scavenging effect, however, oxidation of methionine residues leads to a decrease in the protective effect of A1AT as a protease inhibitor (37). Taggart et al. identified two of the nine methionines as particularly susceptible to oxidation, specifically methionine 358, whose oxidation is already known to result in loss of antielastase activity, and 358 (38).

For this reason, an increasing number of studies reports the development of engineered forms of A1AT, with amino acid substitution at methionine 351 and 358, designed to keep the anti-protease activity, even in a oxidative microenvironment (39, 40).

Additionally, the cysteine residues in A1AT may undergo oxidation, which decreases ROS levels. The oxidation of these cysteine residues can prevent the formation of disulfide bonds, which otherwise might lead to protein misfolding and aggregation. This antioxidant mechanism of A1AT further emphasizes its role in mitigating oxidative stress and maintaining cellular homeostasis (41).

Hydrogen peroxide (H2O2) is another ROS that A1AT can directly reduce. By converting H2O2 into water, A1AT prevents its participation in harmful oxidative reactions. The precise molecular mechanism remains partially elucidated. Hydroxyl radicals are among the most reactive and damaging ROS. A1AT is capable of directly scavenging these radicals, thereby protecting proteins, lipids, and DNA from oxidative damage. This interaction is believed to involve direct electron transfer mechanisms, neutralizing the hydroxyl radicals and attenuating their deleterious effects.

The interaction between AAT and the expression of endogenous antioxidant enzymes such as catalases and superoxide dismutase (SOD) has been known and studied for some time (42).

Recent studies have demonstrated that patients with PI ZZ genotype exhibit reduced expression of catalase and glutathione reductase, indicating an indirect role of alpha-1 antitrypsin (A1AT) in modulating reactive oxygen species (ROS) and reactive nitrogen species (RNS) levels. Specifically, increased levels of H2O2 have been observed in neutrophils isolated from asymptomatic ZZ children, attributed to decreased expression of the antioxidant enzyme catalase. Catalase catalyzes the dismutation of H2O2 into water and oxygen, maintaining intracellular H2O2 concentrations at optimal physiological levels for cellular signaling.

The deficiency of catalase in ZZ- A1ATD patients leads to an accumulation of H2O2, which in turn induces increased expression of glutathione peroxidase (GPx). GPx is a selenium-containing antioxidant enzyme that converts H2O2 and lipid peroxides into water and lipid alcohols, reducing potential oxidative damage. Concurrently, reduced expression of glutathione reductase impairs the regeneration of reduced glutathione (GSH), essential for maintaining the antioxidant activity of GPx.

Furthermore, ZZ-A1ATD patients exhibit altered levels of reactive species: a decrease in superoxide (O2-) and an increase in hydrogen peroxide (H2O2), peroxynitrite (ONOO-), and nitric oxide (NO) are observed. This imbalance suggests that A1AT may play a crucial role in cellular redox regulation, influencing the equilibrium between ROS and RNS. The accumulation of H2O2 and ONOO- can contribute to oxidative and nitrosative stress, potentially leading to cellular damage and immune dysfunction (43).

Superoxide anions (O2−) are primary ROS generated during cellular respiration and various metabolic processes. A1AT significantly reduces the activity of NADPH oxidase in activated neutrophils, the enzyme responsible for transferring electrons from NADPH to molecular oxygen, thereby generating superoxide anions. By inhibiting this enzyme, A1AT decreases the production of superoxides, contributing to a reduction in the overall ROS burden in tissues and helping to limit oxidative damage (44)

Furthermore, A1AT binds to metal ions such as copper (Cu) and iron (Fe), which catalyze ROS production via the Haber-Weiss and Fenton reactions. By sequestering these metal ions, A1AT prevents these catalytic reactions, thus reducing ROS levels. The Haber-Weiss reaction involves the interaction of superoxide anions with hydrogen peroxide to produce hydroxyl radicals, while the Fenton reaction involves the reduction of hydrogen peroxide by ferrous iron, also generating hydroxyl radicals (42, 45).

In addition to the aforementioned role through which A1AT acts with antioxidant action by increasing endogenous antioxidant enzymes, such as superoxide dismutase and catalase, it also exerts indirect mechanisms of protection against oxidative stress. Secondly, A1AT inhibits apoptosis by regulating the activity of caspase-3, a key enzyme in the execution of the apoptotic process. By reducing caspase-3 activation, A1AT prevents programmed cell death, which is often induced by oxidative stress (46).

Finally, A1AT regulates the activity of proteases, enzymes that degrade components of the extracellular matrix. By inhibiting these proteases, A1AT prevents the destruction of the extracellular matrix and the consequent release of additional ROS, thus helping to maintain tissue integrity and limit oxidative damage (47).

These combined mechanisms highlight the importance of A1AT in protecting against oxidative stress and preserving cellular and tissue function.

A1AT influences neutrophils activity by regulating apoptosis in different ways. Apoptosis is a crucial event in the resolution of inflammation, as reduction of activated neutrophils action protects host tissues from injury. A1AT has been shown to reduce *in vitro* apoptosis of neutrophils, by inhibiting the proteasomal degradation of the antiapoptotic protein Mcl-1 (48); conversely, the mutated form of A1AT found in A1AT deficiency increases apoptosis of neutrophils as it accumulates in their cytoplasm, leading to endoplasmic reticulum stress and the expression of proapoptotic signals, including TNF-α (49). Mild inflammation has been demonstrated in the airways of individuals with AATD, despite normal lung function. A tendency for pulmonary function decline in AATD patients has been noted, correlated with a pro-inflammatory phenotype of the lower respiratory tract. Specifically, a threefold increase in neutrophil count, a twofold increase in protease levels, and 2-4 times higher levels of IL-8, IL-6, IL1β, and leukotriene B4 have been observed in the epithelial lining fluid compared to healthy controls (50).

Again, its main effect on cells of the monocyte/macrophages lineage is anti-inflammatory and pro-tolerogenic activity: first of all, *in vitro* studies on human monocytes or total PBMCs (peripheral blood mononuclear cells) evidenced a reduction in the production of proinflammatory cytokines (i.e. IL1-β, TNF-α) as an increase in IL-10 production (51) and IL-1Ra (52). These effects are achieved through different mechanisms: 1) impairing of the ATP-gated P2X7 receptor (one on the main “danger signal” sensors on cells), that activates the NLPR3 inflammasome; a recent work proposed that A1AT could signal through CD36 (a scavenger receptor expressed on monocytes, macrophages and dendritic cells), activating a phospholipase (PLA2) that in turn induces the expression of a small factor with a cholinergic activity that binds acetylcholine receptor in an autocrine manner thus eventually inhibiting the opening of the P2X7 channel (53); 2) increase of intracellular cAMP levels, through activation of adenylate cyclase and induction of cAMP-dependent protein kinase (PKA) (8); 3) increased expression of Nf-κB inhibitor IκB and reduced translocation of Nf-κB to the nucleus (54); 4) regulation of the expression of CD14, a coreceptor for bacterial endotoxin, on cellular membrane (55). As observed in neutrophils, notably the anti-inflammatory effects are described only for long time stimulation, while short term (<2 hours) stimulation results in a synergic effect of A1AT with proinflammatory stimuli (55). The protolerogenic effect of A1AT is evident on dendritic cells (DCs): these cells, at the interface between innate and adaptive immunity, are responsible for antigen presentation to T lymphocytes and priming of naive T cells. In the steady state, DCs patrol tissues and present immature phenotype. After pathogen (or danger) recognition, DCs undergo maturation and express on their membrane a panel of molecules that enable antigen presentation (MHC class II), T lymphocytes costimulation (i.e., CD40, CD80, CD86), and migration to lymph nodes (CXCR4, CCR7). DC maturation in the presence of A1AT leads to a semi-mature profile (smDC), in which there is a reduction in the expression of MHC II, CD40, CD86 compared to LPS stimulated DC, decrease of IL6, increase of IL-10 production (56). This phenotype favors the expansion of regulatory T cells (**Figure 2**).

#### 2.1.2 Effects on adaptive immune system

As for adaptive immunity, there is a general consensus that A1AT reduces the activation of T effector cells and sustains the proliferation of regulatory T cells (Tregs). It is very likely that the effect of A1AT is not direct on lymphocytes, but it is mediated by the alteration in the extracellular milieu (i.e. reduction of IL-1β, TNF-α and IL-6, increase in IL-10) and in DCs maturation. Indeed, there is a lack of evidence of A1AT activity on T lymphocytes *in vitro*, but analysis in murine models of autoimmunity or allotransplant demonstrate that treatment with A1AT determines a reduction of T lymphocytes migration to inflamed tissue and an alteration of Treg versus T effector ratio (57). This effect is also favored from the production of IL-2, that sustain Tregs expansion, in unaffected by A1AT (58).

The potent immunoregulatory role of A1AT may support its therapeutic use in several diseases other than A1AT deficit, particularly in immune- related disorders.

A1AT also has an effect on B lymphocytes, reducing their proliferation and function, as demonstrated in murine models of autoimmune diseases mediated by autoantibodies, such as rheumatoid arthritis, indicating a possible exploitation of A1AT as a therapeutic agent in autoimmune diseases (59) As for the effect on T cells, also the effect of A1AT on B lymphocytes seems to be indirect, mediated by the alterations in the extracellular milieu (**Figure 2**).

### 2.2 Effects on metal and lipid homeostasis

A1AT exhibits the ability to influence metal and lipid homeostasis in addition to its immunomodulatory functions. Observations have indicated a potential association between proteases and iron concentration, suggesting the involvement of antiproteases like A1AT.

In patients with cystic fibrosis (CF), who experience elevated elastase levels in the airways, increased iron and ferritin concentration have been observed in sputum and bronchoalveolar lavage samples (56). Animal studies have demonstrated that the intratracheal instillation of NE is associated with an increase in ferric iron-containing macrophages in the lung airspaces (60).

In individuals with A1AT deficiency, with Z and S polymorphisms, there is a significant accumulation of iron in the liver (61). This suggests that A1AT deficiency may lead to disruptions in iron metabolism and storage.

Studies have also reported significant differences in plasma ferritin concentrations between individuals with different A1AT genotypes. For instance, individuals with the PiZZ genotype, associated with severe A1AT deficiency (A1ATD), have been found to have higher iron and ferritin values compared to those with the PiMM genotype. These differences in iron and ferritin levels cannot be solely attributed to inflammation, as indicated by C-reactive protein levels, suggesting a direct involvement of A1AT in iron metabolism (62).

The ability to influence the iron homeostasis appears to affect the development of neurodegenerative diseases, where disruptions in iron regulation have been observed. Some studies have reported that S or Z polymorphism is present in 25% of patients with anxiety disorders, in 42% of people with bipolar disorder and 10% with pre-existing affective disorders (63, 64).

In addition to iron metabolism, A1AT also affects the metabolism of other metals, such as copper and zinc.

Patients with S and Z polymorphisms have significantly lower values of free copper, not bound to ceruloplasmin, as well as an alteration of zinc homeostasis. In central nervous system, decreased copper stores may result in tissue damage or a reduced response to injury due to a mitochondrial dysfunction. The Z polymorphism has shown an association with demyelinating and hypomyelinating conditions, potentially explaining the higher frequency of demyelinating diseases in individuals with A1AT pathological polymorphisms (65).

Furthermore, A1AT has been implicated in lipid metabolism. Previous research has identified apolipoprotein B-100, a major protein component of low-density lipoprotein (LDL) and very low-density lipoprotein (VLDL), as a binding partner to A1AT. This interaction suggests a potential link and it has been proposed that A1AT may play a role in modulating lipid homeostasis and lipid-related processes through its interaction with apolipoprotein B-100.

Studies have identified the presence of A1AT – LDL (low density lipoprotein) complexes in coronary arteriosclerotic lesions, with A1AT expressed by macrophages in the inner layers of coronary arteries. This suggests that A1AT, produced and oxidized by macrophages, contributes to lipid accumulation in artery wall cells during the early stages of atherogenesis (66).

### 2.3 A therapeutic potential of the use of A1AT

The most appropriate *in vivo* model to understand the anti-inflammatory activity of A1AT administration is enzyme deficiency (A1ATD) condition itself.

Augmentation therapy, which involves the intravenous infusion of purified A1AT protein derived from human plasma, is a treatment approach used to supplement the deficient or dysfunctional A1AT in individuals with A1ATD. By restoring A1AT levels, augmentation therapy aims to slow down the progression of lung disease and reduce the risk of developing complications such as emphysema and increase survival. Real-world data from three national registries (Switzerland, Ireland, and Austria) have recently been published, comprising a total of 615 patients with alpha-1 antitrypsin deficiency (97.7% with the ZZ genotype). The data from this registry demonstrates a survival advantage associated with intravenous A1AT administration, which develops independently and decoupled from the FEV1 decline (67). Alpha-1 antitrypsin (A1AT) augmentation therapy has been shown to impact inflammatory markers such as C-reactive protein (CRP) in patients with Alpha-1 antitrypsin deficiency (AATD). This therapy primarily aims to stabilize lung function and prevent further progression of lung disease, particularly in patients with established emphysema or significant respiratory symptoms.

A1AT augmentation therapy involves intravenous infusions of purified A1AT, which helps to increase the levels of this protein in the blood and lungs. This therapy has been associated with a reduction in inflammation markers, including CRP, which is commonly elevated in inflammatory conditions like COPD. Studies indicate that A1AT therapy can lead to a reduction in systemic inflammation, as measured by lower CRP levels, thus potentially decreasing the overall inflammatory burden on the lungs (68)

Moreover, the reduction in inflammatory markers can also be indicative of the therapy’s effectiveness in mitigating the proteolytic damage caused by neutrophil elastase, which is typically unopposed in AATD patients due to low A1AT levels. By restoring the balance between A1AT and neutrophil elastase, the therapy helps in controlling the chronic inflammatory state that contributes to lung tissue damage (69).

In the lungs of individuals with enzyme deficiency, the overexpression of free neutrophil elastase stimulates the release of leukotriene B4 (LTB4) by interacting with surface receptors on alveolar macrophages. LTB4 is the predominant chemotactic mediator for neutrophils, leading to their recruitment into the lungs (70).

Purified human A1AT has been shown to inhibit neutrophil chemotaxis by reducing NE levels, thus attenuating the recruitment of neutrophils (62). Studies have demonstrated the impact of A1AT therapy, such as with the product Prolastin, on neutrophil chemoattractants. Prolastin administration (60 mg/kg weekly) was found to rapidly reduce LTB4 levels, suggesting its central role in airway inflammation in A1ATD (66).

In a hamster model of fibrosis bleomycin induced, A1AT demonstrated to reduce neutrophil and lymphocyte migration already after 7 days, with a further decrease in cell counts (neutrophils, lymphocytes, and macrophages) after 30 days (71).

Panniculitis is a rare complication of A1ATD that occurs in approximately 0.1% of cases. Nodular lesions with degenerative changes of dermal collagen are the clinical manifestations and the pathogenetic mechanism is likely related to neutrophilic inflammation and an unopposed NE activity (72, 73). In recent years, several cases of panniculitis A1ATD-related successfully treated with augmentation therapy have been reported (74).

Intravenous A1AT infusion is considered the most effective treatment, typically at a dose of 60 mg/kg weekly. The dose and intervals of administration can be adjusted based on the individual’s clinical response (75). Traditionally administered intravenously, a method that has been extensively studied and approved, demonstrating a good safety and efficacy. However, alternative administration routes are being explored to improve convenience and patient adherence to therapy. Inhalation administration, for example, involves using a nebulizer to deliver A1AT directly to the lungs. Preliminary studies suggest that this route could increase A1AT concentration in the airways and reduce systemic side effects, although further data are needed to confirm its efficacy and safety (76, 77).

Another route under investigation is transdermal administration, which involves applying A1AT through the skin demonstrating effective diffusion through the epidermal layers in a concentration- and time-dependent manner. The treatment did not cause any significant morphological alterations or damage to the keratinocyte layers. While promising for enhancing patient convenience, this method still requires further research to fully assess its effectiveness and safety (78).

The use of miRNAs to modulate the expression of the mutant A1AT gene and reduce the associated toxic effects has also been explored. By incorporating miRNA structures into gene therapy agents, it aims to inhibit the production of the mutant Z-AAT protein and promote the production of functional A1AT (79). Therapeutic strategies to boost levels of protective antiproteases such as A1AT in the lung remain an attractive research strategy to limit the damage from excess protease activity. microRNAs are small non-coding RNA molecules that bind specific cognate sequences to inhibit expression of target mRNAs. The inhibition of miRNAs which target the SERPINA1 (A1AT-encoding gene) mRNA represents a novel therapeutic approach for CF inflammation. This could involve the delivery of antagomirs that bind and sequester the target miRNA or target site blockers that bind miRNA recognition elements within the target mRNA to prevent miRNA interaction (80).

These findings highlight the potential of A1AT augmentation therapy to modulate the inflammatory response in A1ATD, reduce neutrophil-mediated damage, and improve clinical outcomes in various manifestations of the disease, including lung inflammation and panniculitis.

#### 2.3.1 Anti-inflammatory effect of A1AT administration in pulmonary disease

##### 2.3.1.1 Anti-inflammatory effect of A1AT administration in COPD

COPD is characterized by chronic inflammation in the airways and lung tissue, leading to airflow limitation and respiratory symptoms. Studies have shown that A1AT administration can have anti-inflammatory effects in COPD by modulating various aspects of the inflammatory response.

By inhibiting neutrophil elastase, A1AT helps to maintain the balance between protease and antiprotease activity in the lungs, reducing tissue damage and inflammation.

In addition, the A1AT ability to modulate the production of pro-inflammatory cytokines and chemokines, such as TNF- alpha, IL- 8, and LTB4 reduces the recruitment and activation of inflammatory cells in COPD.

In a study conducted on transgenic mice expressing extremely low levels of A1AT and exhibiting to daily cigarette smoke for up to 6 months, the use of augmentation treatment (Prolastin 20 mg every 48 hours) resulted in a significant reduction in lavage neutrophils and macrophages, approximately of 75% and 50% respectively after 6 months. Additionally, the administration of A1AT reduced airspace size (mean linear intercept [Lm]) by 63% compared with smoke-exposed not treated (22).

Furthermore, the study demonstrated that A1AT therapy suppressed the increase of TNF-alpha smoke-mediated in serum. This observation prompted the authors to consider whether the protective mechanism of A1AT involves not only the direct inhibition of NE activity but also an anti-inflammatory effect that prevents the release of TNF- alpha and subsequent infiltration of inflammatory cells.

Subsequent research on murine model revealed that A1AT can inhibit both macrophages metalloproteinase (MMP-12) production and the release of TNF-alpha from alveolar macrophages, thereby reducing smoke-induced inflammation and the influx of inflammatory cells (23).

One proposed mechanism for the increased production of MMP-12 is the activation of proteinase-activated receptor-1 (PAR-1) by serum constituents, such as plasmin/plasminogen and thrombin, which leak into the lung after smoke exposure. Gearing and colleagues suppose that A1AT may prevent the release of MMP-12 and TNF-alpha by inhibiting the activity of both thrombin and plasmin (81). Overall, these findings indicate that augmentation therapy has the potential to attenuate the inflammatory response in the lungs of mice exposed to cigarette smoke. The reduction in lavage neutrophils, macrophages, and airspace size, along with the suppression of TNF- alpha and MMP-12, suggests a multi-faceted anti-inflammatory effect of A1AT treatment.

The clinical-functional impact of A1AT therapy in patients with emphysema associated with A1ATD has been extensively demonstrated through numerous clinical trials. The RAPID and RAPID Open Label Extension (RAPID-OLE) studies represent the largest multicenter randomized placebo-controlled trials conducted on this therapy. The RAPID-OLE study, published in The Lancet in 2015, confirmed the efficacy of augmentation therapy on lung density, measured by computed tomography (CT densitometry), over a total observation and treatment period of four years. Already in the initial RAPID-RCT study, a significant slowdown in lung density loss was observed compared to placebo, indicating a marked reduction in the progression of emphysema. This reduction is crucial as it is associated with less lung tissue destruction and an improved prognosis for patients with AATD. Moreover, the RAPID-OLE study demonstrated that the lung density lost during the two years of placebo treatment is not recovered with the introduction of A1PI, thus highlighting the importance of early therapeutic intervention with a highly purified A1PI product (12, 82).

Despite recent studies favoring radiological monitoring, several studies have evaluated the effect of therapy on lung function. In particular, a systematic review of 5632 patients concluded that augmentation therapy has a slight effect in reducing the decline in lung function, showing a 23% slowdown in the decline of forced expiratory volume in one second (FEV1) (13.4 mL/year, 95% CI 1.5-25.3 mL/year) in treated patients compared to the placebo group (83).

Additionally, a longitudinal study on 96 patients with severe AATD analyzed the rate of FEV1 decline before and after the initiation of weekly augmentation therapy, showing a slower decline in FEV1 in patients with mild airflow obstruction during treatment (84).

Regarding long-term clinical trial data on patient outcomes such as the frequency of exacerbations, quality of life, need for lung transplantation, and mortality, these remain more limited (85, 86).

The effect of AAT therapy in reducing exacerbations has been described in patients with severe COPD. In an observational study, the rate of exacerbations decreased from 3-5 infections/year before therapy to 0-1 infections/year after the initiation of therapy. Although randomized studies have not shown an overall effect on the frequency of exacerbations, a *post-hoc* analysis of a clinical trial reported a decrease in their severity (87)

Overall, these results demonstrate that A1PI augmentation therapy offers significant benefits in slowing the progression of emphysema and improving clinical outcomes for patients with AATD, underscoring the importance of timely and continuous treatment.

##### 2.3.1.2 Anti-inflammatory effect of A1AT administration in cystic fibrosis

Cystic fibrosis, a genetic disorder that primarily affects the lungs and digestive system, is caused by mutations in the cystic fibrosis transmembrane conductance regulator (CFTR) gene, which leads to the production of defective CFTR proteins. CFTR is responsible for regulating the movement of salt and water in and out of cells, and its dysfunction results in the production of thick, and sticky mucus in various organs. In the lungs, the abnormal mucus obstructs the airways and impairs the clearance of bacteria, leading to chronic respiratory infections, inflammation, and progressive lung damage.

CF is characterized by sustained neutrophil recruitment and neutrophil dominated inflammation from a very young age, then the damage is also mediated by NE activity (13, 14).

Several studies have investigated the potential of A1AT as a therapeutic intervention.

McElvaney and colleagues were the first to suggest the use of aerosolized A1AT therapy. They demonstrated a complete inhibition of NE activity within the respiratory epithelial lining fluid (ELF), after only one week of aerosolization of 1.5mg/kg A1AT every 12 hours. Importantly, A1AT therapy did not interfere with the ability of neutrophil-mediated killing of Pseudomonas aeruginosa, a common pathogen in CF (13).

These findings have been subsequently supported by other studies. Griese et al. showed a decrease in sputum NE activity, neutrophil count, levels of pro-inflammatory cytokines (IL- 8, IL-1beta, TNF- alpha and LTB4) and the numbers of Pseudomonas aeruginosa after 2 and 4 weeks aerosolized A1AT treatment. However, they did not observe any significant improvement in lung function (84).

A phase II trial aimed at assessing the clinical efficacy of aerosolized A1AT demonstrated a significant improvement in the time to the first pulmonary exacerbation and the total number of exacerbations. Importantly, no significant differences in adverse events were reported, indicating that the treatment was well tolerated (15).

These studies highlight the potentials of aerosolized A1AT therapy in reducing NE activity, neutrophil-mediated inflammation, and the presence of bacterial pathogens in the lungs of individuals affected by CF.

##### 2.3.1.3 Immunomodulatory effects in resistant asthma and atopic status

Asthma is a chronic inflammatory condition of the airways that leads to airway hyperresponsiveness, bronchoconstriction, and respiratory symptoms. This inflammation involves the activation of various immune cells and the release of inflammatory mediators, which contribute to the characteristic features of the disease.

Eosinophilic inflammation is the most common type of inflammation seen in asthma. Eosinophils play a significant role in allergic and eosinophilic asthma. However, in some individuals with asthma, a different pattern of inflammation may be present. In Th2 low phenotype a neutrophilic inflammation may drive the inflammatory process (88).

Several studies have reported a prevalence of asthma ranging from 20 to 50% in individuals with homozygous (ZZ) and heterozygous (SZ) A1ATD (16).

It is hypothesized that augmentation therapy could improve inflammation, reduce bronchial hyperresponsiveness, and prevent the development of chronic airway changes in these patients.

Data from of the National Heart, Lung, and Blood Institute Registry suggest that the augmentation treatment is not more effective in reducing the decline of forced expiratory volume in one second (FEV1) in the groups with asthma compared to the group without. However, subgroup analysis indicated better efficacy in patients with FEV1 between 35 to 49% category (16).

In a case report published in 2008, Blanco et al. described a successful treatment outcome with A1AT in a Caucasian 27-year-old woman affected by Widal triad with a severe and resistant asthma and an MZ phenotype. The patient exhibited a statistically significant improvement in lung function, with an increase in FEV1 from 43% to 52%. The treatment regimen involved an initial dose of 60 mg/kg/week for two weeks, followed by 120 mg/kg/biweekly for seven months. The therapy was well-tolerated without any drug-related adverse effects. A1AT treatment reduced the number of emergency consultations and hospital admissions, decreased the need for steroid therapy, and progressively improved the patient’s quality of life (17).

The association of A1ATD with allergies is estimated at 29%, with elevated IgE levels observed in 17% of cases. Elevated IgE levels are significantly associated with asthma symptoms and a history of allergies (16).

Additionally, studies on nasal lavage fluid have shown a close relationship between A1AT and eosinophil activation in patients with allergic rhinitis, particularly after allergen stimulation (89). Immunoelectron microscopy studies conducted by Johansson et al. demonstrated the presence of A1AT in the specific granules of eosinophils (90). These findings suggest a possible role for A1AT in the treatment of atopic asthma and other allergic diseases, possibly through its immunomodulatory effects on eosinophils.

Furthermore, He and coworkers sustained a protective role for A1AT in mast cell associated disease, including allergy, due to its inhibitory effect on IgE-induced histamine release from mast cells (91).

However, to date, no studies have demonstrated the usefulness of augmentation therapy in treating atopic manifestations.

In summary, while there is evidence suggesting the potential benefits ofA1AT therapy in asthma, particularly in individuals with lower FEV1 levels, further research is needed to establish its efficacy and optimal application in the treatment of atopic manifestations and allergic diseases.

#### 2.3.2 Usefulness in autoimmune diseases

##### 2.3.2.1 Rheumatoid arthritis

Rheumatoid arthritis (RA) is a chronic autoimmune disease characterized by a complex and dynamic immunopathogenesis. The development and progression of RA involve the intricate interplay of various cell types, cytokine, and molecular pathways.

In the last recent years, the association between low serum levels of A1AT and the development of RA has been identified, particularly in relation to specific A1AT genotypes. RA patients with the A1ATD PiMZ phenotype have been found to exhibit van increased prevalence and higher titers of anticitrullinated peptide autoantibodies (ACPA), indicating a distinct subset with greater disease severity (92). Furthermore, the presence of antibodies targeting carbamylated A1AT (Ca-A1AT), identified as a potential target of anti-carbamylated (anti CarP) antibodies, has been detected in the synovial compartment, suggesting a contribution to synovial inflammation (93).

There has been growing interest in other autoantibodies that recognize post-translationally modified proteins. In this context, Colasanti and coworkers have identified to homocysteinylated A1AT (Hcy-A1AT) in 54.4% of seropositive RA, but not in seronegative cases, indicating their potential relevance in the disease (94).

Dysregulated activation of neutrophils is known to contribute to the pathogenesis of RA, although the precise mechanisms governing their activation remain largely unknown (131).

In various experimental animal models, the use of neutrophil elastase inhibitor has been shown to reduce the incidence and severity of collagen-induced arthritis (CIA) and mitigate histologically demonstrated damage to articular cartilage (24, 95).

In this regard, Griemstein and colleagues have demonstrated the potential utility of A1AT in RA using a murine model. Administration of human A1AT (hA1AT) either as a protein or through a recombinant adeno-associated virus-mediated (rA1AT) gene therapy resulted in reduced levels of serum and autoantibodies against bovine type II collagen and mouse collagen II. This treatment significantly delayed disease onset and modified disease progression at both macroscopic and histopathological levels (95).

##### 2.3.2.2 Gout arthritis

Gout arthritis is characterized by a deposition of uric acid crystals in the joint, which triggers an inflammatory response. The pathogenesis of gout arthritis involves several key factors but IL- 1beta is an important cytokine associated with the progression of the disease. Uric acid stimulates human blood monocytes to increase the production and the release of IL- 1beta, and the activation is mediated by an enzyme, PR3.

A1AT is known to inhibit the function of PR3, reducing the activity of IL-1 beta and consequently decreasing the inflammatory response in gout (96).

In animal studies, transgenic mice expressing A1AT have shown increased transcription and secretion of IL-1 receptor agonist (IL-1Ra) which has anti-inflammatory effects. IL-1Ra acts as a counterbalance to IL-1beta, mitigating its inflammatory actions and promoting a more balanced immune response (96).

##### 2.3.2.3 Systemic vasculitis

Granulomatosis with polyangiitis (GPA), microscopic polyangiitis (MPA) and eosinophilic granulomatosis with polyangiitis (EGPA) are associated with antineutrophil cytoplasmic antibodies (ANCA). ANCAs can be direct against directly opposed proteinase 3 (PR3) and myeloperoxidase (MPO) which are targets of antiproteinase activity of A1AT. In the light of this, consideration deficiency in A1AT is a possible pathogenic cofactor in ANCA-associated vasculitis (AAV) (97).

Although the clinical impact of AATD in AAV is not yet fully understood, the American Thoracic Society/European Respiratory Society recommends assessing A1AT levels in cases of anti-PR3 vasculitis (98). Studies have shown that the Z allele, associated with A1ATD, is present in 5-27% of individuals with GPA (98). Rahmattulla et al. found a significant association between both the S and Z alleles and AAV, including perinuclear ANCA or MPO-ANCA (99). To date, there are no studies showing the efficacy and safety of augmentation therapy in systemic vasculitis but there are case reports on its successful use in cutaneous vasculitis in individuals with A1ATD, particularly with PiZZ phenotype (18).

##### 2.3.2.4 Fibromyalgia

Fibromyalgia (FM) is a chronic disorder characterized by widespread musculoskeletal pain, fatigue, sleep disorders, and heightened sensitivity to pressure or touch. The exact cause is still unknown, and it is generally considered a complex disorder that arises from a combination of genetic, environmental, and psychological factors.

Recent evidence suggests a potential association between FM and A1ATD. In according to an epidemiological study, the prevalence of MZ, SZ and ZZ polymorphisms is 2- 4 times higher in FM than in general population. The A1ATD can be considered as a predisposing factor for the development of early and severe onset of FM in an individual with low A1AT serum levels (7% MZ, 0.5% SZ and 0.2% ZZ) (100). Additionally, three reported cases have shown raid and persistent control of fibromyalgia symptoms in individuals treated with intravenous A1AT (101, 102).

Furthermore, a blinded immunohistochemical study of skin biopsies revealed a significant increase in the number of mast cells in the dermis of fibromyalgia patients compared to healthy subjects (103). Considering this finding, exogenous A1AT could potentially act as a mast cells stabilizer and neutralizer of mast cells mediators in FM. However, larger trials are required to determine the clinical efficacy of this therapy in FM patients.

#### 2.3.3 Usefulness in graft-versus-host disease

Acute GVHD is a common complication in patients undergoing hematopoietic stem cell transplantation, it occurs in 20-80% and approximately 40% of patients are refractory to first line therapy. Currently, there is no established standard treatment for acute and steroid-refractory GVHD (SR-aGVHD) (19, 104, 105).

Studies have shown that A1AT has the potential to induce tolerance in pre-clinical models of GVHD, particularly gastrointestinal steroid-refractory seems to be an optimal setting. A1AT has been found to modulate the cytokine environment, promote a tolerogenic shift of dendritic cells, and favor the development of effector T-cells towards regulatory T-cell (19).

In a phase I/II open-label single-center study, A1AT was administered as salvage therapy to 12 patients with SR-aGVHD. Clinical manifestation improved in 8 of 12 subjects of which 4 had a complete response, in absence of relevant toxicities (20).

These positive findings were further supported by a multicenter clinical study involving 40 patients with SR-aGVHD. The patients received intravenous A1AT twice weekly for 4 weeks as a first-line treatment. By day 28, the 35% of subjects achieved a complete response, and at day 60, 73% of the responses were sustained without the need for additional immunosuppression (21).

Notably, Giannoni et al. demonstrated a favorable response to A1AT in patients with advanced-stage gut SR-GVHD, including those who had previously failed other treatments for SR-GVHD (19). Gastrointestinal response was observed in 61% of patients, with a median time to best response of 21 days (19).

In a multicenter proof-of-concept trial, 30 patients high-risk with high-risk of developing SR-GVHD received twice-weekly infusions of A1AT for a total of 16 doses. Their outcomes were compared to a control group of 90 high-risk patients from the Mount Sinai Acute GVHD International Consortium (MAGIC) study. The treatment was well tolerated with minimal side effects, but it did not significantly reduce the incidence of SR GVHD compared to the control group. Based on these findings, it can be concluded that the specific dose and schedule of A1AT used in this trial did not effectively decrease the occurrence of SR GVHD (106).

Further research is needed to explore optimal dosing strategies and identify potential predictors of treatment response in SR-aGVHD.

#### 2.3.4 Usefulness in diabetes mellitus

Type 1 Diabetes Mellitus (DM1) is a chronic condition characterized by the autoimmune destruction of insulin-secreted beta cells in the pancreas, severe insulin deficiency may result in chronic hyperglycemia with many complications. Interestingly, individuals with DM1 exhibit lower plasma concentrations and decreased activity of A1AT compared to other people (107). These findings were subsequently also confirmed in Type 2 DM (DM2) (108). A1AT inhibits the activity of neutrophil elastase and other proteases, reducing the inflammatory response. This is crucial in diabetes, where chronic inflammation is a significant contributing factor to insulin resistance and beta-cell dysfunction (68, 109). The process of beta-cell death appears to be enhanced by A1AT deficiency (28, 109). A1AT has also been shown to inhibit the nuclear factor kappa-light-chain-enhancer of activated B cells (NF-κB) pathway, which plays a critical role in the inflammatory process. By reducing NF-κB activation, A1AT can decrease the expression of pro-inflammatory genes, thus mitigating inflammation-associated insulin resistance (110). A1AT can protect pancreatic beta cells from apoptosis induced by inflammatory cytokines and oxidative stress. This preservation of beta-cell function is essential in maintaining insulin production and secretion in diabetic patients (111). Furthermore, A1AT can reduce endoplasmic reticulum (ER) stress in beta cells, which is a common problem in diabetes that leads to cell dysfunction and death. By alleviating ER stress, A1AT helps maintain beta-cell integrity and function (112).

By introducing additional A1AT through gene delivery using a recombinant adeno-associated virus, researchers were able to observe a significant reduction in insulitis and prevent the development of hyperglycemia in non-obese diabetic mice (30).

A notable finding in this context is the potential of A1AT to extend the survival of transplanted islet cells and regulate the immune response in a mouse model of islet cell transplantation (27, 29, 113, 114).

The underlying mechanism of action appears to involve an increase in insulin secretion that is dependent on glucose but also potentiated by glucagon-like peptide-1 and forskolin. Furthermore, A1AT has been shown to protect and rescue INS- 1E, a diabetic cell line, from apoptosis induced by TNF-alpha. It also reduces the levels of apoptosis induced by IL-1beta and IFN-gamma (115).

In summary, A1ATD contributes to beta-cell death in diabetes. Using A1AT through gene delivery it is possible to reduce inflammation and prolong the survival of transplanted islet cells and modulate immune response. The mechanism of action involves increased glucose-dependent insulin secretion, potentiation by glucagon-like-peptide-1 and forskolin, protection against apoptosis in diabetic cell lines, and reduction of apoptosis induced by inflammatory factors.

By reducing systemic inflammation, A1AT can improve insulin signaling pathways. This results in better glucose uptake by tissues and improved glycemic control (116).

Finally, A1AT antioxidant properties can neutralize reactive oxygen species (ROS), reducing oxidative stress that contributes to both beta-cell dysfunction and insulin resistance.

#### 2.3.5 Usefulness in infectious disease

Several studies have emphasized the therapeutic potential of A1AT in the control of infection and the pathogenicity of microbes. Specifically, research has revealed antimicrobial and anti-inflammatory properties of A1AT that could provide defense against bacterial lung infections (13).

Chan et al. discovered a higher occurrence of A1AT in individuals with chronic lung disease caused by rapidly growing mycobacteria (RGM). The incidence was approximately 1.6 times more frequent compared to the general US population. This finding suggested a potential role of A1AT in defense against such pathogens. Subsequently, the researchers demonstrated in laboratory experiments a suppression of mycobacterium abscessus infection of monocyte-derived macrophages by up to 65% (115).

Interestingly, A1AT replacement therapy administered to patients with A1ATD has shown potential in reducing the risk of respiratory infections. As previously discussed, aerosolized A1AT has a positive impact on neutrophil-mediated killing of P. Aeruginosa in Cystic Fibrosis (13). In a rat model of chronic P. Aeruginosa lung infection, aerosolized Prolastin significantly decreased bacterial colonial counts, although it did not have a direct bactericidal effect in laboratory tests. This observation suggests a potential use of aerosolized A1AT as a non-antibiotic adjunct in the treatment and control of infections in CF (26).

The role of A1AT extends beyond antimicrobial protection of the airways, as demonstrated in the context of enteropathogenic Escherichia coli (EPEC) infection. It appears that A1AT interferes with secretion protein B (EspB), which is responsible for pore formation on the host cell membrane, thus responsible for hemolysis of host red blood cells. It would appear that EPEC-mediated hemolysis is strongly reduced by A1AT, in a concentration-dependent way (117).

Other studies have also described a similar mechanism involving the inhibition of human immunodeficiency virus type 1 (HIV-1). The virus enters cells through the interaction of its glycoproteins, gp120 and gp41, with cell surface proteases. A1AT has been hypothesized to disrupt the interaction between gp120 and these proteases, thus interfering with the viral entry process (118).

Munch et al. identified a virus inhibitory peptide (VIRIP) within the C-proximal region of A1AT, which acts as an important inhibitor of a wide variety of HIV-1 strains, including those resistant to current antiretroviral drugs. The mechanism seems to be mediated to gp41 fusion peptide, it has antiviral therapeutic potential (119).

Another highlighted mechanism involves the ability of A1AT to suppress NF-κB activation, although the exact mechanism of action remains still unknown (120).

The clinical implications of these laboratory findings are exemplified by the description of unusually rapid declines in CD4+ T cell concentrations in HIV-infected individuals with A1ATD (121).

#### 2.3.6 A1AT and SARS- CoV-2

As observed in the case of the Middle East Respiratory Syndrome (MERS) caused by the coronavirus (122), A1AT has been suggested to that A1AT plays a role in protecting against SARS-CoV-2 infection.

**Figure 3:**
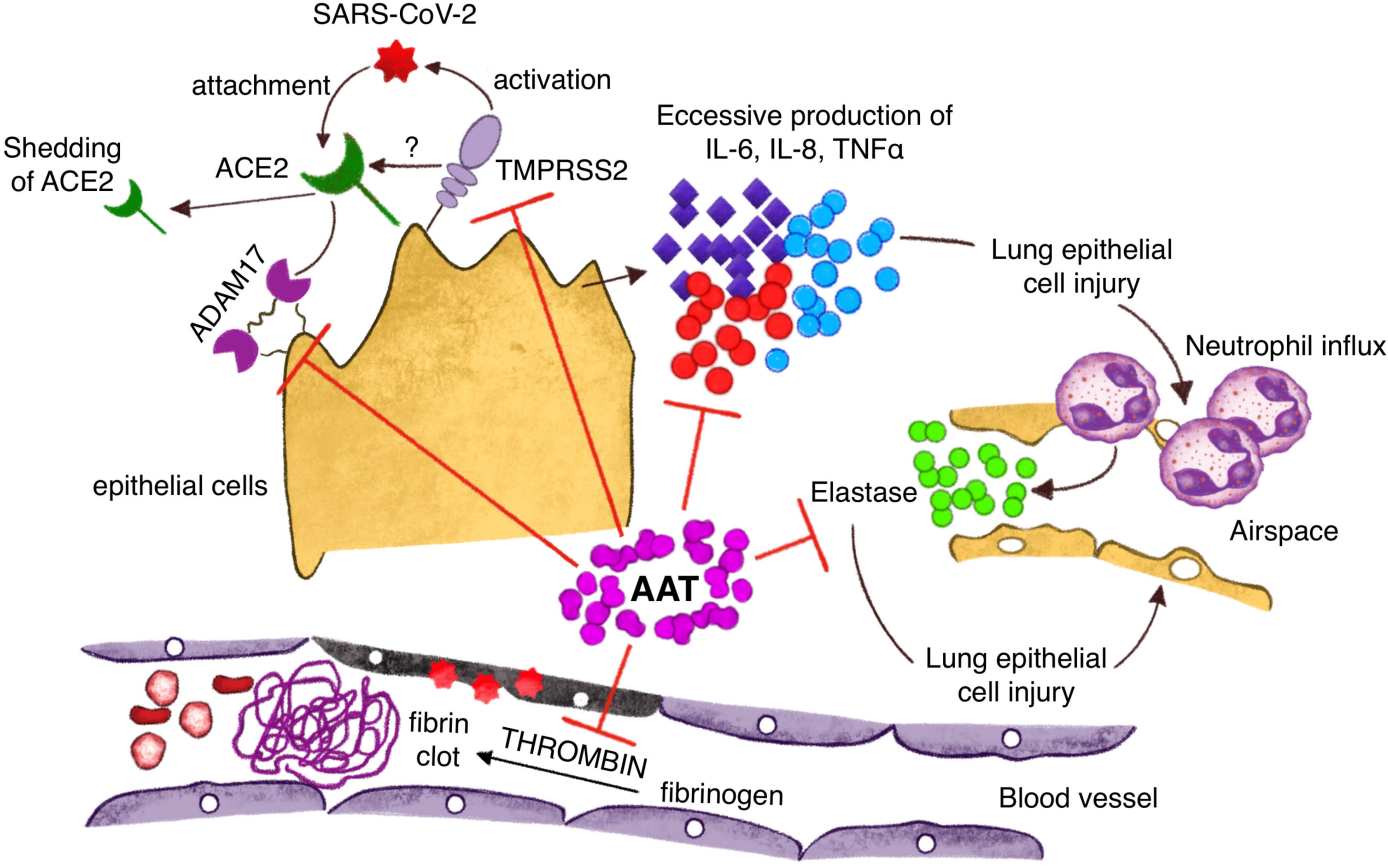
Protective mechanisms of A1AT against Sars Cov2 infection: inhibition of virus entry into the cells through action on TMPRSS2 and ADAM 17; A1AT antagonizes inflammatory cytokines with a reduction of neutrophils pulmonary influx; A1AT inhibits lung epithelial cell injury through direct action on neutrophil elastase and vascular damage inhibiting fibrin clots formation.

A1AT appears to inhibit viral infection by targeting two crucial proteases involved in the pathophysiology of the virus: the transmembrane serine protease 2 (TMPRSS2) and the ADAM17 (123, 124). Additionally, A1AT inhibits the activity of inflammatory molecules such as IL-8, TNF-α, and NE. TMPRSS2 is essential for facilitating the infection process, as it cleaves the spike protein of SARS-CoV-2, enabling the virus to bind to its cell surface receptor, angiotensin converting enzyme 2 (ACE2), and gain entry into cells. ADAM17 involved in the release of ACE2, IL-6R, and TNF-α. ACE2 is a key component for the balance of the renin angiotensin system, inflammation, vascular permeability, and pulmonary homeostasis (**Figure 3**) (122, 123). Neutrophil elastase (NE) plays a significant role in the pathogenesis of SARS-CoV-2, particularly in the development of severe lung disease such as acute respiratory distress syndrome (ARDS). During SARS-CoV-2 infection, neutrophils are recruited to the lungs and release NE, which contributes to tissue damage and inflammation. This process is exacerbated by the formation of neutrophil extracellular traps (NETs), web-like structures composed of DNA, NE, and other antimicrobial factors. NETs can trap and kill pathogens but also cause local obstruction in the lungs, promoting mucin overproduction and furthering inflammation and thrombosis, contributing to severe lung injury and poor outcomes in COVID-19 patients​ (125–127). In addition to its direct damaging effects, NE can enhance the activation of the immune response, contributing to a cytokine storm, a dangerous hyper-inflammatory condition seen in severe COVID-19 cases. Clinical interventions targeting NE, such as NE inhibitors like sivelestat, are being explored for their potential to mitigate lung damage and improve outcomes in COVID-19-induced ARDS​ (128). Understanding the dual role of NE in pathogen defense and tissue damage provides insights into potential therapeutic strategies for managing severe COVID-19. By targeting NE and its associated pathways, it may be possible to reduce lung injury and improve survival rates in critically ill patients. ​ Clinical evidence suggests that A1AT levels may be important in determining the outcomes of COVID-19 (6, 129, 130). An Italian analysis of the A1ATD population highlighted a higher frequency of SARS-CoV-2 infection compared to national data, approximately 3.8% (130). Another recent clinical analysis with a cohort of 40 COVID-19 patients demonstrated increased A1AT levels. Typically, the rise in A1AT is directly proportional to the increase in IL-6, indicating an anti-inflammatory function. Consequently, the authors propose that A1AT augmentation therapy should be considered and investigated as a potential treatment for COVID-19. In this context, it is plausible to consider A1AT as a protective host factor against COVID-19, not only decreasing SARS-CoV-2 entry, but also protecting from the main clinical complications, such as acute inflammation and acute respiratory insufficiency (67). However, further research is needed to fully understand these aspects.

## 3 Conclusion

Considering the cumulative evidence supporting the beneficial effects of A1AT in chronic inflammatory diseases, it is important to conduct comprehensive clinical studies to ascertain its true clinical efficacy in patient populations affected by each specific disease.

While *in vitro* studies and animal models have provided positive indications, it is crucial to validate these findings in human subjects to establish the relevance and applicability of A1AT augmentation treatment.
